# The lower motor neuron homunculus

**DOI:** 10.1093/brain/awac310

**Published:** 2022-08-27

**Authors:** John Ravits, Julia Stack

**Affiliations:** Department of Neurosciences, School of Medicine, University of California at San Diego, MC 0624, La Jolla, CA 92093, USA; Drawbones, Inc, Seattle, WA 98117, USA

## Abstract

Penfield’s motor homunculus anthropomorphizes the cerebral level of motor control, the upper motor neuron. However, it leaves the cranial and spinal motor neurons unrepresented. Here Ravits and Stack redress the imbalance by presenting a lower motor neuron homunculus.

***Penfield’s motor homunculus anthropomorphizes the cerebral aspect of the motor system, the upper motor neuron. However, it leaves the cranial and spinal aspects, the lower motor neuron or final common pathway, to be so represented. Here we redress the imbalance by presenting a lower motor neuron homunculus. He is shown juxtaposed to Penfield’s motor homunculus in anatomic proportion to highlight key comparisons.***

Penfield and Boldrey published in *Brain* the original motor homunculus of man in 1937 based on results obtained from neurophysiological stimulation along the motor cortex in patients.^1^ The name ‘homunculus’ means ‘little man’. Preformationism in Pythagoras time in 500s Bc and alchemy in the 16th century also previously had used the term. Penfield’s motor homunculus displayed pictorially the shape and size of somatic regions in proportion to their cortical representation. The key concept captured by this was that the organization of motor cortex is ‘somatotopic’, and their innovation was to represent this visually. Personifying neuronal anatomy gave ‘a visual image [based on] the size and sequence of cortical area [and thus] the size of the parts of this grotesque creature were determined [by] the apparent perpendicular extent of the representation of each part’.^1^ Penfield published a more pleasing version of the motor homunculus in a 1950 monograph *The Cerebral Cortex of Man: A Clinical Study of Localization of Function* in which somatic regions of the homunculus were overlaid on coronal sections through the motor cortex. Ms H. P. Cantlie was the graphic illustrator of both versions—the original 1937 article did not acknowledge her but the Preface to the 1950 monograph did.

The pictorial characterization of the Penfield homunculus was a culmination in understanding organization of motor cortex that extended over a century of research involving clinicians, anatomists, histologists, and neurophysiologists from around the world in the 19th and early 20th centuries (reviewed in Finger^2^). Key contributors were Hitzig and Fritsch, who used electrical stimulation to demonstrate the role of motor cortex in movement and further studies by Ferrier. Jackson characterized the somatotopic nature of the motor anatomy of the cortex through studies of epileptic patients. Betz identified the unique giant-sized neurons in layer V of the motor cortex that are now named after him. Brodmann and Campbell characterized the cytoarchitecture of the brain, including motor cortical regions. By 1917, Leyton and Sherrington had outlined somatotopic anatomy of the motor cortex in primates and Foerster, in his Lecture to the Royal Society of Medicine reproduced in *Brain*, did so for humans in 1936.

The motor homunculus unexpectedly captured the imagination of readers and over time achieved meme-like status. But the figure has not been without criticisms.^3–5^ Concerns have been voiced that the homunculus oversimplified and caricaturized motor anatomy rather than presented its extraordinary complexity. The figure suggested brain representation involved direct motor innervation rather than ‘functional engrams’; the border at the Rolandic sulcus between precentral and postcentral gyri was distinct rather than variable, overlapping and discontinuous; circuits were conceptualized as being direct rather than capturing their complexity, with numerous inputs and connections (now called the ‘connectome’). Methodological criticisms have concerned the lack of rigour in mapping the motor cortex including quality control, histology, validation, transparency of stimulation parameters, reproducibility, data transformation, statistics, and even data sharing. As a result, Roux *et al*.^6^ have recently re-mapped and revised the motor cerebral cartograph. Recently, social criticisms have also been raised concerning male-dominance as manifested by lack of involvement or acknowledgment of women or study of female somatotopic anatomy^5^—Wright recently created a *her*munculus to do this.^7^ In sum, while Penfield’s motor homunculus is imperfect, still today he ‘is a [beloved] metaphor for the complex neurological mechanisms that we strive to comprehend in their entirety … [and is] a brilliant *aide-mémoire*'.^3^

One criticism of Penfield’s motor homunculus that has not been raised is that he is incomplete, representing only the cerebral level of motor control, the cortico-motor neurons, and leaving spinal and cranial motor neurons—the part of the motor system that executes motor work—unrepresented. In parallel to, but separate from, elucidation of the organization of motor cortex, clinicians, anatomists, histologists and neurophysiologists from around the world also elucidated fundamental understanding of spinal and cranial motor neurons over the same century of research (reviewed in Barbara and Clarac^8^ and Clarac and Barbara^9^). Bell and Magendie identified that the anterior roots control motor contractility, while Deiters and Remak surmised the continuity of motor cells and their processes including the fibres of the anterior horn exiting the spinal cord and further distinguished between motor and sensory function. The giant cells in the anterior horn of the spinal cord were characterized in detail by von Kölliker who ascribed motor properties to them. Duchenne in his *Physiologie des Mouvements* formalized segmental anatomy known as the myotomes. Cajal used spinal motor neurons as a model for his newly emerging concepts of neurons and occasionally applied the term ‘motor neuron’ to them. Sherrington identified integrative properties of motor neurons and referred to them as ‘the final common pathway’ and along with his mentee Liddell, formulated the concept of ‘the motor unit’ as the fundamental element of motor physiology comprising a motor neuron and its axon and muscle fibres.

Gowers proposed the most enduring concept that brought together the discoveries about brain and spinal cord motor functions that were made during this time (reviewed in Phillips and Landau^10^). In the first editions of his *Manual of Diseases of the Nervous System* that started in 1886, he stated that neurologists should consider ‘the whole motor path, from cortex of the brain to the muscles [and we] may consider it as composed of two segments, an upper and a lower'. By the time he published the third edition in 1899, the neuron theory was transforming the view of the nervous system and Gowers changed the language from upper segment and lower segment to upper motor neuron and lower motor neuron. Upper motor neuron comprises cerebrospinal elements that terminate in the grey substance to connect to the lower motor neuron, which comprises spinomuscular elements. He states that ‘this conception of the motor path [as composed of these two neuron levels] conduces to clearer ideas of many facts of disease, and it is important to grasp it firmly. We shall see, for instance, that diseases involving any part of a neuron produce similar effects, however diverse their nature; while there is a fundamental difference between the effects of disease of the two neurons.’ Gower’s formulation remains today still essentially unaltered as a fundamental axiom of localization in clinical neurology—‘the little old synecdoche that works'.^10^

In this context, Penfield’s motor homunculus anthropomorphizes the upper motor neuron, the cerebral aspect of the motor system, but leaves the lower motor neuron, the final common pathway, to be so represented. To redress this, we present here a lower motor neuron homunculus, who is shown juxtaposed to his upper motor neuron partner to highlight their relative proportions and differences (Fig. 1). The dimensions are based on data from studies of both gross and histological anatomy (Table 1 and Supplementary material) allowing the artist (J.S.) aesthetic leeway, including omission of the tongue. The height of the lower motor neuron homunculus is based on the average rostral-caudal measurements of the brainstem, cervical, thoracic, and lumbosacral cords. The height of the upper motor neuron homunculus is based on the average coronal measurement along the motor gyrus from the Sylvian fissure to the cingulate gyrus. In both, the length of the arms is subtracted from overall height. The relative proportion of the two heights is approximately 3:1. The girths of the somatic regions in the lower motor neuron homunculus are based on average alpha motor neuron densities in CNSs. The girths of the somatic regions in the upper motor neuron homunculus are based on lateral to medial span (estimated to be one-third for face, one-third for arm and one-third for leg) rather than based on Betz cell density, which is generally uniform along the motor cortex. To the best of our knowledge, this represents the first depiction of a lower motor neuron homunculus, and he is juxtaposed to the celebrated cerebral motor partner.

**Figure 1:**
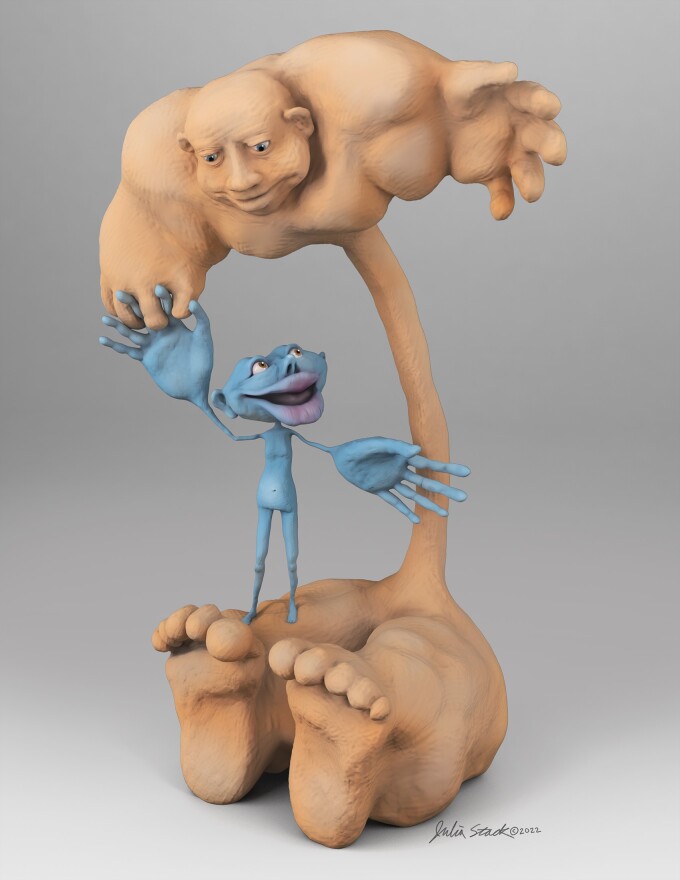
**The lower motor neuron homunculus and his celebrated upper motor neuron partner.** The lower motor neuron homunculus (*right*) is juxtaposed to the upper motor neuron homunculus (*left*) and they are drawn in anatomic proportion.

**Table 1:** Anatomic comparison of upper motor neuron and lower motor neuron, and their representations in the motor homunculi

Anatomical feature	Upper motor neuron	Lower motor neuron
Gross anatomy	Primary motor cortex (Brodmann area 4 or M1)	Cranial motor nuclei and spinal anterior horns
Cyto-architecture	Betz cells, which are layered in layer V, are relatively uniformly distributed along the motor cortex—this is not directly represented in girth of somatic regions of the homunculus	Alpha motor neurons, which are stacked in columns in brainstem and Rexed lamina IX of spinal cord, have relative densities of 3:3:1:5 in hypoglossal nucleus, cervical, thoracic, and lumbar anterior horns—this is represented as girths of somatic regions
Somatotopic organization (head to toes)	Lateral to medial along cortex to organize into motor tracts	Rostral to caudal to organize into cranial nerves and motor roots (myotomes)
Anatomic dimensions	Span along motor gyrus from Sylvian fissure to cingulate gyrus is 12 cm per hemisphere and this is represented in the homunculus by overall height (arm length is estimated to be one-third and subtracted from overall height.) Note disproportionate length of face and arms.	Height of motor column from pons to sacral cord is 45 cm (brainstem 4.5–6; cervical cord, 10–13 cm; thoracic cord, 20–25 cm; and lumbosacral cord, 5–8 cm) and this is represented in the homunculus by overall height (arm length is subtracted from overall height.) Note disproportionate length of trunk.

For further information, see the Supplementary material.

## Supplementary Material

awac310_Supplementary_Data
