# Correction to: The lower motor neuron homunculus

**DOI:** 10.1093/brain/awac383

**Published:** 2022-11-16

John Ravits, Julia Stack. The lower motor neuron homunculus. *Brain*. 2022(11):3727–3729. https://doi.org/10.1093/brain/awac310

The authors apologize for a citation error in the fifth paragraph. The text should read: Gower’s formulation remains today still essentially unaltered as a fundamental axiom of localization in clinical neurology—‘the little old synecdoche that works’.^10^

