# Supplementary material for: The lower motor neuron homunculus
Source: Brain. 2022 Aug 27;145(11):3727–9. doi: 10.1093/brain/awac310 (PMC9679163; doi:10.1093/brain/awac310)
Supplement: awac310_Supplementary_Data [file awac310_supplementary_data.pdf]

LMN Homunculus Supplementary Material: Methods Used in Determining Homunculi Sizes

1. Overview
Heights are based on measurements of length and somatic girths are based on motor neuron density. The homunculi are drawn as close to actual measurements as reasonable, allowing the artist (JK) esthetic leeway.

2. Demographics
All nervous systems had been acquired with an Investigational Review Board and Health Insurance Portability and Accountability Act compliant informed consent process in the Benaroya Research Center ALS CNS repository as previously published [1].

3. Length Measurements Used to Estimate Homunculi Height
Methods: Fresh brains and spinal cords were measured at autopsy using pliant tape measures. Cortical determinations were measured from the Sylvian fissure to the vertex and vertex to cingulate gyrus [2]. Rostral-caudal brainstem and spinal cord determinations were estimated for midbrain, pons, medulla, upper cervical segment above the cervical enlargement, cervical enlargement, thoracic segment, lumbosacral enlargement, and sacral segment below the enlargement. Up to 11 determinations were obtained and averaged.

Results (Supplementary Materials page 2): The average lateral-medial spans along the motor gyrus from Sylvian fissure to vertex is ~10-12 cm and vertex to cingulate gyrus is ~2-3 cm. These are comparable to published data [2]. The average brainstem and spinal cord rostral-caudal spans are: pons, ~3-3.5 cm; medulla, ~1.5-2.5; cervical cord, 10-13 cm; thoracic cord, ~20-25 cm; and lumbosacral cord, ~ 5-8 cm [see Figure on page 2]. For homunculus characterizations, arm lengths (estimated at the cortex to be ~1/3 overall span and cervical enlargement) are not calculated in estimations of height.

4. Neuron "Particle" Counting Used to Estimate Girth of Somatic Regions of LMN Homunculus
Comment: Girths of the homunculi are based on motor neuron density, the technical challenges of which have been recently reviewed [3]. For the lower motor neuron homunculus, these are based on measurements of alpha motor neuron density as previously published [1]; for the upper motor neuron homunculus, these reflect what are presumed to be density of Betz cells at the cortex relatively commensurate to length, recognizing this complexity [4].
Methods: We used the control data from our previously described series [1]. There were 8 control nervous systems, 7 male and 1 female. The mean age was 63 years (range 38-80 years). None had known neurological diseases. Post-mortem intervals averaged 5.25 hours (range 2.0-12 hours). The published method is restated here for convenience.
Histological Sampling: We used 6 µm thick sections from formalin-fixed paraffin-embedded tissues stained with cresyl violet acetate. We studied 4 neuraxis levels: hypoglossal nucleus at the mid-medulla, mid-cervical spinal enlargement, mid-thoracic spinal region, and mid-lumbar spinal enlargement. We standardized rostral-caudal sampling of the hypoglossal nucleus by its relation to the medial longitudinal fasciculus and by the morphology of the 4th ventricle [5]. We standardized rostral-caudal sampling of spinal cord regions by choosing middle segments—the cervical region has uniform counts between C4-C8 [6,7], the lumbar region has uniform neuron counts between L3-S2 [8,9,10], and we assume the mid-thoracic region has uniform counts. Since motor neuron presence varies from one histological section to another [6,11], we evaluated a total of 8 sections from each neuraxis level, choosing every 10th section for a sampling interval of 60 µm from a series of 84 consecutive sections spanning 504 µm, a sampling technique modified from Method 1 of Tomlinson et al [11]. We separately imaged each side of the spinal anterior horns and hypoglossal nucleus under 50X with a Leica DM2500 microscope with a Spot Insight 4 digital camera and Spot Advanced V4.5.7 software (Diagnostic Instruments, Inc., Sterling Heights, MI) and stored each image in Tagged-Image File Format.
Counting: To meet the realities of the counting task, we devised a method of counting neuron "particles" to index relative neuron presence rather than determining absolute neuron counts [3,12]—stereology technologies do not readily apply to the motor neurons [13] and automated particle counting software was inefficient, experience reported by others [14]. Our method had 3 observers independently count motor neuron particles in each image file according to the following criteria learned in training sessions to standardize counting: neurons were located in the anterior portion of the anterior horns of the spinal cord segments or in the hypoglossal nucleus [5], had relatively deep Nissl staining, were generally multi-concave, and were larger than 25 µm in diameter in cervical and lumbar regions and larger than 15-20 µm in thoracic and hypoglossal regions. Both right and left sides were counted. Counting neurons in the perihypoglossal nuclei, the intermediolateral cell column, and Clarke's column was avoided. Because we were not concerned with pathological change or cell shrinkage, we did not correct for split cell error [15,16].
Statistics and Calculations: We compiled particle counts in a spreadsheet and collated and processed them with simple statistics.
Results (Supplementary Materials Page 3): Hypoglossal nucleus in medulla = 32 motor neuron particles/cross-sectional area/side; cervical level = 33 motor neuron particles/cross-sectional area/side; thoracic level = 10 motor neuron particles/cross-sectional area/side; lumbar level 48 motor neuron particles/cross-sectional area/side. Neuron counts, contrary to other reports [7.8,17], did not have pronounced age-related changes. The ratio of medulla:cervical:thoracic:lumbar= ~3:3:1:5.

5, References

[1] Ravits J, Laurie P, Fan Y, Moore DH. Implications of ALS focality: rostral-caudal distribution of lower motor neuron loss postmortem. Neurology. 2007 May 8;68(19):1576-82. doi: 10.1212/01.wnl.0000261045.57095.56. PMID: 17485644.
[2] Ono M, Kubik S, Abernathey C. Atlas of the cerebral sulci. Stuttgart: Verlag, 1990:151–161.
[3] Ferrucci M, Lazzeri G, Flaibani M, Biagioni F, Cantini F, Madonna M, Bucci D, Limanaqi F, Soldani P, Fornai F. In search for a gold-standard procedure to count motor neurons in the spinal cord. Histol Histopathol. 2018 Oct;33(10):1021-1046. PMID: 29537481.
[4] Rivara CB, Sherwood CC, Bouras C, Hof PR. Stereologic characterization and spatial distribution patterns of Betz cells in the human primary motor cortex. Anat Rec A Discov Mol Cell Evol Biol. 2003 Feb;270(2):137-51. doi: 10.1002/ar.a.10015. PMID: 12524689.
[5] Olszewski J, Baxter D: Cytoarchitecture of the Human Brain Stem (2nd Edition). S. Karger, Basel (Switzerland), 1982, plates VIII-XX (pp 26-38) and pp 62-63.
[6] Elliott HC: Studies on the motor cells of the spinal cord. I. Distribution in the normal human cord. Am J Anat 1942;70:95-117.
[7] Yuan H, Ke M, Goto N, Goto J, Suzuki K. Morphometric evaluation of the human cervical motoneurons. Okajimas Folia Anat Jpn. 2000 Mar;76(6):277-84. doi: 10.2535/ofaj1936.76.6_277. PMID: 10774225.
[8] Irving D, Rebeiz JJ, Tomlinson BE. The numbers of limb motor neurones in the individual segments of the human lumbosacral spinal cord. J Neurol Sci. 1974 Feb;21(2):203-12. doi: 10.1016/0022-510x(74)90072-0. PMID: 4443821.
[9] Kawamura Y, O'Brien P, Okazaki H, Dyck PJ. Lumbar motoneurons of man II: the number and diameter distribution of large- and intermediate-diameter cytons in "motoneuron columns" of spinal cord of man. J Neuropathol Exp Neurol. 1977 Sep-Oct;36(5):861-70. doi: 10.1097/00005072-197709000-00010. PMID: 894329.
[10] Kawamura Y, Dyck PJ. Lumbar motoneurons of man: III. The number and diameter distribution of large- and intermediate- diameter cytons by nuclear columns. J Neuropathol Exp Neurol. 1977 Nov;36(6):956-63. doi: 10.1097/00005072-197711000-00007. PMID: 925720.
[11] Tomlinson BE, Irving D, Rebeiz JJ. Total numbers of limb motor neurones in the human lumbosacral cord and an analysis of the accuracy of various sampling procedures. J Neurol Sci. 1973 Nov;20(3):313-27. doi: 10.1016/0022-510x(73)90193-7. PMID: 4587145.
[12] Coggeshall RE, Lekan HA. Methods for determining numbers of cells and synapses: a case for more uniform standards of review. J Comp Neurol. 1996 Jan 1;364(1):6-15. doi: 10.1002/(SICI)1096-9861(19960101)364:1<6::AID-CNE2>3.0.CO;2-9. Erratum in: J Comp Neurol 1996 May 20;369(1):162. PMID: 8789272.
[13] Schmitz C, Hof PR. Design-based stereology in neuroscience. Neuroscience. 2005;130(4):813-31. doi: 10.1016/j.neuroscience.2004.08.050. PMID: 15652981.
[14] Stephens B, Guiloff RJ, Navarrete R, Newman P, Nikhar N, Lewis P. Widespread loss of neuronal populations in the spinal ventral horn in sporadic motor neuron disease. A morphometric study. J Neurol Sci. 2006 May 15;244(1-2):41-58. doi: 10.1016/j.jns.2005.12.003. Epub 2006 Feb 17. PMID: 16487542.
[15] Abercrombie M: Estimation of nuclear population from microtome sections. Anat Rec. 1946 Feb;94:239-47. doi: 10.1002/ar.1090940210. PMID: 21015608.
[16] Offord K, Ota M, Oenning RF, Dyck PJ. Method of morphometric evaluation of spinal and autonomic ganglia. J Neurol Sci. 1974 May;22(1):65-71. doi: 10.1016/0022-510x(74)90054-9. PMID: 4598264.
[17] Tomlinson BE, Irving D. The numbers of limb motor neurons in the human lumbosacral cord throughout life. J Neurol Sci. 1977 Nov;34(2):213-9. doi: 10.1016/0022-510x(77)90069-7. PMID: 925710.

.

Homunculi Height Determinations

Supplemental Table 1: Motor Cortex, Cranial, and Spinal Lengths (cm)										
CNS#	Age	Sex	Dx	Motor Cortex	Pons	Med-ulla	Cervical Cord (Upper)	Cervical (main)	Thor-acic Cord	Lumbo-sacral Cord
25	53	M	SALS	10	3.5	NA	NA	NA	NA	NA
28	49	F	SALS	10.5	3.5	NA	NA	NA	NA	NA
37	57	M	Control	10.7	3.5	NA	NA	NA	NA	NA
39	77	M	Control	10	NA	NA	NA	NA	21	5
40	76	F	Control	9.5	3.5	1.5	NA	10	24.5	5.5
41	81	M	Control	9.5	3.5	NA	NA	11	24	7
42	61	M	Control	10	3.2	1.5	2.5	10	21	8
43	74	M	SALS	9.5	NA	1.8	2	10	25	5
44	80	F	Control	9.5	3.1	NA	NA	10	23	6
46	51	F	SALS	10		NA	NA	10.5	20	5
47	65	F	SALS	10.7	3.5	2.5	2.5	10.5	24	6

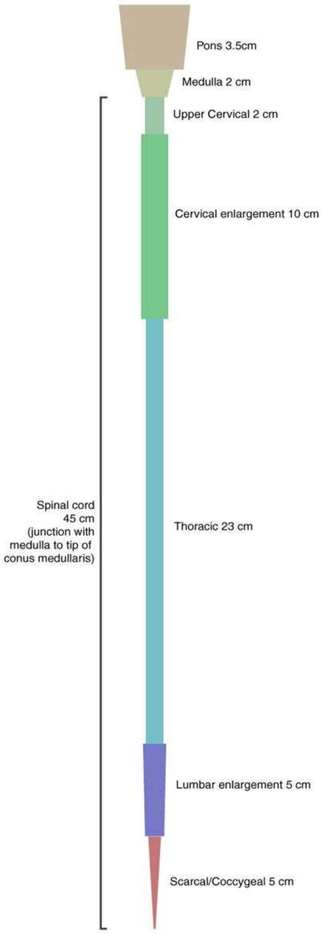

Lower Motor Neuron Counts (reproduced from supplementary reference 1)

Supplementary Table 2: Demographics			
CNS#	Dx	Age	Sex
7	Control	61	M
10	Control	78	M
19	Control	80	F
20	Control	38	M
23	Control	77	M
26	Control	49	M
31	Control	67	M
37	Control	57	M

Supplementary Table 3: Summary				
Region	Observer: JR	Observer: YF	Observer: PL	AVERAGE
**Medulla AVE**	33	36	25	32
**Medulla SD**	12	11	9	
**Cervical AVE**	30	37	32	33
**Cervical SD**	18	19	18	
**Thoracic AVE**	8	13	9	10
**Thoracic SD**	3	4	4	
**Lumbar AVE**	43	53	48	48
**Lumbar SD**	10	10	9	

Supplementary Table 4: Particle Counts

Region	Observer: JR	Observer: YF	Observer: PL
**Medullas**	10	19	8
**Medullas**	12	19	10
**Medullas**	14	20	11
**Medullas**	15	20	12
**Medullas**	16	21	12
**Medullas**	17	22	13
**Medullas**	18	22	13
**Medullas**	18	23	13
**Medullas**	19	24	14
**Medullas**	19	24	14
**Medullas**	19	24	14
**Medullas**	20	24	15
**Medullas**	20	25	15
**Medullas**	20	25	15
**Medullas**	21	26	15
**Medullas**	21	26	16
**Medullas**	21	26	16
**Medullas**	22	26	16
**Medullas**	22	26	16
**Medullas**	22	27	16
**Medullas**	22	27	16
**Medullas**	22	27	17
**Medullas**	23	27	17
**Medullas**	23	28	18
**Medullas**	23	28	18
**Medullas**	24	28	18
**Medullas**	24	28	18
**Medullas**	24	28	19
**Medullas**	25	28	19
**Medullas**	25	28	19
**Medullas**	25	28	19
**Medullas**	25	29	19
**Medullas**	26	29	19
**Medullas**	26	29	19
**Medullas**	26	29	19
**Medullas**	26	29	20
**Medullas**	26	29	20
**Medullas**	26	29	20
**Medullas**	26	30	20
**Medullas**	27	30	21
**Medullas**	27	30	21
**Medullas**	28	31	21
**Medullas**	28	31	21
**Medullas**	28	31	21
**Medullas**	29	31	21
**Medullas**	29	31	21
**Medullas**	29	31	21
**Medullas**	29	32	21
**Medullas**	30	32	22
**Medullas**	30	32	22
**Medullas**	30	32	22
**Medullas**	30	32	22
**Medullas**	30	32	22
**Medullas**	31	32	22
**Medullas**	31	33	23
**Medullas**	31	33	23
**Medullas**	31	33	23
**Medullas**	31	33	23
**Medullas**	31	33	23
**Medullas**	32	33	23
**Medullas**	32	33	24
**Medullas**	32	33	24
**Medullas**	32	34	24
**Medullas**	32	34	24
**Medullas**	33	34	25
**Medullas**	33	34	25
**Medullas**	34	34	25
**Medullas**	34	35	25
**Medullas**	34	35	25
**Medullas**	34	35	25
**Medullas**	34	35	26
**Medullas**	35	36	26
**Medullas**	35	36	26
**Medullas**	35	36	26
**Medullas**	36	36	26
**Medullas**	36	36	26
**Medullas**	36	36	26
**Medullas**	37	37	27
**Medullas**	37	37	27
**Medullas**	37	37	27
**Medullas**	37	37	28
**Medullas**	37	38	28
**Medullas**	37	38	28
**Medullas**	37	39	28
**Medullas**	38	39	28
**Medullas**	38	39	29
**Medullas**	38	39	29
**Medullas**	38	40	29
**Medullas**	38	40	29
**Medullas**	39	40	29
**Medullas**	39	41	30
**Medullas**	39	41	31
**Medullas**	39	41	31
**Medullas**	40	41	32
**Medullas**	40	41	32
**Medullas**	40	42	33
**Medullas**	41	42	33
**Medullas**	41	43	33
**Medullas**	41	43	34
**Medullas**	41	44	34
**Medullas**	42	44	34
**Medullas**	42	44	35
**Medullas**	42	45	36
**Medullas**	43	46	36
**Medullas**	43	46	36
**Medullas**	43	46	37
**Medullas**	43	47	37
**Medullas**	44	47	37
**Medullas**	44	47	37
**Medullas**	45	48	38
**Medullas**	47	49	39
**Medullas**	47	49	39
**Medullas**	47	50	39
**Medullas**	48	50	41
**Medullas**	48	50	41
**Medullas**	50	51	42
**Medullas**	54	55	42
**Medullas**	56	55	43
**Medullas**	57	57	43
**Medullas**	57	60	44
**Medullas**	60	62	45
**Medullas**	63	64	46
**Medullas**	65	66	54
**Medullas**	67	69	*20
**Medullas**	67	77	*24
**AVE**	33.48	36.08	25.40650407
**SD**	11.5182884	10.7497937	9.052521973

	Observer: JR	Observer: YF	Observer: PL
**Cervical**	2	4	3
**Cervical**	2	6	3
**Cervical**	2	6	3
**Cervical**	3	7	3
**Cervical**	3	7	3
**Cervical**	4	7	4
**Cervical**	4	7	5
**Cervical**	5	7	5
**Cervical**	5	8	6
**Cervical**	6	8	6
**Cervical**	6	8	6
**Cervical**	6	8	6
**Cervical**	7	9	6
**Cervical**	7	9	6
**Cervical**	8	9	7
**Cervical**	8	9	7
**Cervical**	8	9	7
**Cervical**	8	10	8
**Cervical**	9	11	9
**Cervical**	9	11	9
**Cervical**	10	12	10
**Cervical**	11	12	11
**Cervical**	11	13	11
**Cervical**	11	14	12
**Cervical**	13	17	13
**Cervical**	15	17	17
**Cervical**	16	18	18
**Cervical**	16	22	18
**Cervical**	18	27	19
**Cervical**	20	28	21
**Cervical**	20	29	22
**Cervical**	20	31	24
**Cervical**	22	32	24
**Cervical**	22	32	26
**Cervical**	23	33	26
**Cervical**	23	34	26
**Cervical**	24	35	27
**Cervical**	24	36	27
**Cervical**	25	37	28
**Cervical**	25	37	28
**Cervical**	25	37	29
**Cervical**	26	37	29
**Cervical**	26	38	29
**Cervical**	27	38	30
**Cervical**	27	38	30
**Cervical**	27	39	30
**Cervical**	28	39	31
**Cervical**	29	39	32
**Cervical**	29	39	33
**Cervical**	29	40	34
**Cervical**	30	40	34
**Cervical**	30	40	34
**Cervical**	31	40	34
**Cervical**	31	41	35
**Cervical**	32	41	35
**Cervical**	32	41	35
**Cervical**	33	42	35
**Cervical**	33	42	36
**Cervical**	33	42	36
**Cervical**	33	43	37
**Cervical**	34	43	37
**Cervical**	35	43	38
**Cervical**	37	43	38
**Cervical**	37	44	40
**Cervical**	38	44	40
**Cervical**	38	44	41
**Cervical**	38	44	41
**Cervical**	40	45	42
**Cervical**	40	47	42
**Cervical**	41	47	42
**Cervical**	41	48	42
**Cervical**	41	48	42
**Cervical**	42	49	43
**Cervical**	42	49	43
**Cervical**	43	49	44
**Cervical**	43	50	44
**Cervical**	44	51	46
**Cervical**	44	51	46
**Cervical**	45	52	48
**Cervical**	45	52	49
**Cervical**	46	52	49
**Cervical**	47	53	50
**Cervical**	47	54	51
**Cervical**	47	56	51
**Cervical**	48	56	52
**Cervical**	49	57	53
**Cervical**	50	58	53
**Cervical**	51	58	54
**Cervical**	52	59	54
**Cervical**	52	59	56
**Cervical**	54	60	56
**Cervical**	56	60	57
**Cervical**	56	60	59
**Cervical**	57	61	60
**Cervical**	58	62	60
**Cervical**	58	63	61
**Cervical**	61	64	61
**Cervical**	62	68	62
**Cervical**	64	70	62
**Cervical**	68	73	66
**Cervical**	74	84	70
**AVE**	30.06930693	36.65346535	31.91089109
**SD**	17.90489175	19.21532495	18.35979249

	Observer: JR	Observer: YF	Observer: PL
**Thoracic**	2	4	2
**Thoracic**	2	5	3
**Thoracic**	3	6	3
**Thoracic**	3	6	4
**Thoracic**	3	7	4
**Thoracic**	3	7	4
**Thoracic**	4	7	4
**Thoracic**	4	7	4
**Thoracic**	4	7	5
**Thoracic**	4	7	5
**Thoracic**	4	7	5
**Thoracic**	4	7	5
**Thoracic**	4	8	5
**Thoracic**	4	8	6
**Thoracic**	4	8	6
**Thoracic**	4	8	6
**Thoracic**	5	8	6
**Thoracic**	5	8	6
**Thoracic**	5	8	6
**Thoracic**	5	9	6
**Thoracic**	5	9	6
**Thoracic**	5	9	6
**Thoracic**	5	9	6
**Thoracic**	5	9	6
**Thoracic**	5	9	6
**Thoracic**	5	9	6
**Thoracic**	5	9	6
**Thoracic**	5	9	6
**Thoracic**	5	9	6
**Thoracic**	5	9	6
**Thoracic**	5	10	7
**Thoracic**	5	10	7
**Thoracic**	5	10	7
**Thoracic**	5	10	7
**Thoracic**	5	10	7
**Thoracic**	6	10	7
**Thoracic**	6	10	7
**Thoracic**	6	10	7
**Thoracic**	6	10	7
**Thoracic**	6	10	7
**Thoracic**	6	10	7
**Thoracic**	6	10	7
**Thoracic**	6	11	7
**Thoracic**	6	11	8
**Thoracic**	6	11	8
**Thoracic**	6	11	8
**Thoracic**	6	11	8
**Thoracic**	6	11	8
**Thoracic**	6	11	8
**Thoracic**	7	11	8
**Thoracic**	7	11	8
**Thoracic**	7	11	8
**Thoracic**	7	11	8
**Thoracic**	7	12	8
**Thoracic**	7	12	8
**Thoracic**	7	12	8
**Thoracic**	7	12	8
**Thoracic**	7	12	8
**Thoracic**	7	12	8
**Thoracic**	7	12	8
**Thoracic**	7	12	8
**Thoracic**	7	12	8
**Thoracic**	7	12	8
**Thoracic**	7	12	9
**Thoracic**	8	13	9
**Thoracic**	8	13	9
**Thoracic**	8	13	9
**Thoracic**	8	13	9
**Thoracic**	8	13	9
**Thoracic**	8	13	9
**Thoracic**	8	13	9
**Thoracic**	8	14	9
**Thoracic**	8	14	9
**Thoracic**	8	14	9
**Thoracic**	8	14	9
**Thoracic**	8	15	9
**Thoracic**	8	15	9
**Thoracic**	8	15	9
**Thoracic**	8	15	10
**Thoracic**	8	15	10
**Thoracic**	8	15	10
**Thoracic**	8	16	10
**Thoracic**	8	16	10
**Thoracic**	9	16	10
**Thoracic**	9	16	10
**Thoracic**	9	16	10
**Thoracic**	9	16	10
**Thoracic**	9	16	11
**Thoracic**	9	16	11
**Thoracic**	9	16	11
**Thoracic**	9	16	11
**Thoracic**	9	16	11
**Thoracic**	10	16	11
**Thoracic**	10	16	11
**Thoracic**	10	16	11
**Thoracic**	10	17	12
**Thoracic**	10	17	12
**Thoracic**	10	17	12
**Thoracic**	10	17	12
**Thoracic**	10	17	13
**Thoracic**	11	17	13
**Thoracic**	11	17	13
**Thoracic**	11	17	13
**Thoracic**	11	17	13
**Thoracic**	11	17	13
**Thoracic**	11	18	14
**Thoracic**	11	18	14
**Thoracic**	11	19	14
**Thoracic**	11	19	15
**Thoracic**	12	19	15
**Thoracic**	12	20	15
**Thoracic**	12	20	15
**Thoracic**	12	20	15
**Thoracic**	12	21	16
**Thoracic**	12	21	16
**Thoracic**	12	21	16
**Thoracic**	13	21	16
**Thoracic**	13	21	16
**Thoracic**	14	22	17
**Thoracic**	15	22	17
**Thoracic**	16	23	19
**Thoracic**	17	24	22
**AVE**	7.57377049	12.9918	9.155738
**SD**	2.85226216	4.32513	3.656807

	Observer: JR	Observer : YF	Observer: PL
**Lumbar**	16	31	29
**Lumbar**	21	33	30
**Lumbar**	22	35	32
**Lumbar**	22	35	33
**Lumbar**	23	36	34
**Lumbar**	23	36	34
**Lumbar**	23	37	34
**Lumbar**	24	37	35
**Lumbar**	25	37	35
**Lumbar**	26	39	35
**Lumbar**	26	39	35
**Lumbar**	27	40	36
**Lumbar**	28	40	36
**Lumbar**	29	41	36
**Lumbar**	32	41	36
**Lumbar**	32	41	36
**Lumbar**	32	41	37
**Lumbar**	33	42	37
**Lumbar**	33	42	37
**Lumbar**	34	42	37
**Lumbar**	34	43	38
**Lumbar**	34	44	38
**Lumbar**	35	44	39
**Lumbar**	35	44	39
**Lumbar**	35	44	39
**Lumbar**	35	44	40
**Lumbar**	35	45	40
**Lumbar**	35	45	40
**Lumbar**	36	45	41
**Lumbar**	37	45	41
**Lumbar**	37	46	41
**Lumbar**	37	46	41
**Lumbar**	38	46	42
**Lumbar**	38	46	42
**Lumbar**	38	46	43
**Lumbar**	38	46	43
**Lumbar**	38	47	43
**Lumbar**	39	47	43
**Lumbar**	39	49	43
**Lumbar**	39	49	43
**Lumbar**	39	49	43
**Lumbar**	39	49	43
**Lumbar**	39	49	44
**Lumbar**	40	49	44
**Lumbar**	40	49	44
**Lumbar**	40	49	44
**Lumbar**	41	49	44
**Lumbar**	41	50	44
**Lumbar**	42	50	44
**Lumbar**	42	50	45
**Lumbar**	42	50	45
**Lumbar**	42	50	45
**Lumbar**	42	50	45
**Lumbar**	42	50	45
**Lumbar**	43	51	45
**Lumbar**	43	51	45
**Lumbar**	43	51	45
**Lumbar**	43	51	46
**Lumbar**	43	51	46
**Lumbar**	44	52	46
**Lumbar**	44	52	46
**Lumbar**	44	52	46
**Lumbar**	44	52	46
**Lumbar**	44	53	47
**Lumbar**	45	53	47
**Lumbar**	45	53	47
**Lumbar**	45	53	47
**Lumbar**	45	54	48
**Lumbar**	45	54	48
**Lumbar**	45	54	48
**Lumbar**	46	54	48
**Lumbar**	46	55	48
**Lumbar**	46	55	48
**Lumbar**	46	55	49
**Lumbar**	46	55	49
**Lumbar**	46	56	49
**Lumbar**	47	56	50
**Lumbar**	47	56	50
**Lumbar**	48	56	50
**Lumbar**	48	57	50
**Lumbar**	48	57	50
**Lumbar**	48	57	50
**Lumbar**	48	57	51
**Lumbar**	48	57	51
**Lumbar**	48	58	52
**Lumbar**	48	58	52
**Lumbar**	48	58	52
**Lumbar**	48	58	52
**Lumbar**	48	58	52
**Lumbar**	49	58	53
**Lumbar**	49	59	54
**Lumbar**	49	59	54
**Lumbar**	49	59	54
**Lumbar**	50	59	55
**Lumbar**	50	60	55
**Lumbar**	51	60	55
**Lumbar**	51	61	55
**Lumbar**	51	61	55
**Lumbar**	51	61	56
**Lumbar**	52	62	57
**Lumbar**	52	62	57
**Lumbar**	53	62	57
**Lumbar**	53	62	57
**Lumbar**	53	63	58
**Lumbar**	53	63	58
**Lumbar**	53	63	58
**Lumbar**	53	64	59
**Lumbar**	53	64	59
**Lumbar**	53	65	59
**Lumbar**	54	66	59
**Lumbar**	54	66	60
**Lumbar**	54	67	60
**Lumbar**	54	67	60
**Lumbar**	54	67	62
**Lumbar**	54	68	62
**Lumbar**	55	68	62
**Lumbar**	55	68	62
**Lumbar**	56	69	62
**Lumbar**	56	69	62
**Lumbar**	57	69	63
**Lumbar**	57	69	63
**Lumbar**	58	69	64
**Lumbar**	59	71	64
**Lumbar**	60	71	66
**Lumbar**	62	72	66
**Lumbar**	62	73	67
**Lumbar**	65	74	68
**Lumbar**	66	76	69
**AVE**	43.4140625	53.3203	48.078125
**SD**	10.086654	9.90569	9.17296201
